# Does Responsiveness to Basic Tastes Influence Preadolescents’ Food Liking? Investigating Taste Responsiveness Segment on Bitter-Sour-Sweet and Salty-Umami Model Food Samples

**DOI:** 10.3390/nu13082721

**Published:** 2021-08-07

**Authors:** Ervina Ervina, Valérie L. Almli, Ingunn Berget, Sara Spinelli, Julia Sick, Caterina Dinnella

**Affiliations:** 1 Nofima, Norwegian Institute of Food, Fisheries and Aquaculture Research, 1433 Ås, Norway; valerie.lengard.almli@nofima.no (V.L.A.); ingunn.berget@nofima.no (I.B.); 2 Department of Chemistry, Biotechnology and Food Science (KBM), The Norwegian University of Life Science, 1433 Ås, Norway; 3 Department of Agriculture, Food, Environment and Forestry (DAGRI), University of Florence, Via Donizetti 6, 50411 Florence, Italy; sara.spinelli@unifi.it (S.S.); julia.sick@unifi.it (J.S.); caterina.dinnella@unifi.it (C.D.)

**Keywords:** taste intensity, individual differences, food preferences, suppression, bitterness, sourness, warning sensations, remote testing, children

## Abstract

The objective of this study was to investigate the relationships between taste responsiveness and food liking in preadolescents. Model food samples of grapefruit juice (GF) and vegetable broth (VB) modified with four additions of sucrose and sodium chloride, respectively, were employed. Intensity perception for sweetness, sourness, and bitterness were measured in GF while saltiness and umami were measured in VB. The children (*N* = 148) also completed food choice, familiarity, stated liking and neophobia questionnaires. The test was conducted at school, with instructions provided remotely via video call. Four segments were defined differing in basic taste responsiveness. Segments and sucrose concentrations significantly affected liking for GF, while no significant effect of segments and sodium chloride concentrations occurred on liking for VB. An increasing sucrose concentration was positively associated with liking for GF only in the segment with low responsiveness to bitter and sour tastes. No significant differences across segments were found for food choice, familiarity, stated liking, and neophobia. Conclusively, relationships between taste responsiveness and liking are product and basic taste-dependent in addition to being subject-dependent. Strategies to improve acceptance by using sucrose as a suppressor for warning sensations of bitterness and sourness can be more or less effective depending on individual responsiveness to the basic tastes.

## 1. Introduction

Taste has been shown to be the most important motive in children’s food choice and acceptance, independently of age. This was reported in children aged 12–13 years [1], 4–6 years [2], and in infants less than one year old [3,4]. Taste is recognized as one of the drivers of children’s food preferences and intake [5,6,7,8]. According to Reed and Knaapila [9], sweet, salty, and umami tastes could initiate liking, while in contrast bitter and sour tastes were associated with food aversion. The low intake of fruits and vegetables in preadolescent children may be related to their taste preferences, due to the presence of bitter and sour tastes in fruits and vegetables [1,10]. On the other hand, children prefer foods characterized by a high content of fat, sugar, and salt [11,12,13], which can contribute to increasing the risk of childhood obesity [14,15]. Sweetness is one of the basic tastes strongly associated with children’s food acceptance [4,16,17,18] while bitterness is usually associated with food rejection since this taste is biologically linked with poisonous or toxic substances [18,19,20] although not all bitter compounds are toxic. Preferences for sour tastes in children provide equivocal results. Children aged 9–14 years prefer to consume fruit drinks with low sourness intensity, indicating a negative association between sour taste and children’s food liking [21]. However, a previous study demonstrated that sour taste from citric acid in a water solution sample was the most liked compared to other basic tastes investigated in 11-year-old children [11].

Children’s food preferences may be associated with their taste intensity perception, also known as taste responsiveness [22,23,24,25]. Taste responsiveness varies across individuals, and has been reported both in adults [26,27] and in preadolescents [11,28,29]. Individual differences in taste perception have been reported to be correlated with genetics [30,31,32]. PROP (6-*n*-prophylthiouracil) has been considered as a general marker for perception of a variety of chemosensory experiences [8,33]. Subjects with high intensity perception of PROP bitterness generally have heightened responses to other basic tastes as well [8,11,34]. Some of the studies did find a relationship between responsiveness to PROP and vegetable intakes such as reported by Bell and Tepper [35], indicating that 4–5-year-old children with low bitter responsiveness have a higher vegetable intake for broccoli, black olives and cucumber compared to children with high responsiveness. PROP intensity perception moderates the relationships between food consumption pattern and Body Mass Index (BMI) in 8–10-year-old children [36], where processed foods intake positively associated with body composition in non-tasters, but not in PROP-tasters. Moreover, responsiveness to bitter taste (quinine) significantly decreased the acceptance of grapefruit juice in 9–11-year-old children [28]. Inconclusive results were observed for saltiness, as Liem [37] reported that there was no strong relationship between saltiness sensitivity measured by detection threshold and preferences for salty foods in children, while Kim and Lee [38] reported that 12–13-year-old children with a higher detection threshold of saltiness have a higher liking for stew and soup. In regard to umami taste, the results from a previous study demonstrated that high umami threshold in 11-year-old children correlated to the increased of stated liking for bitter-umami foods [11]. Moreover, responsiveness to umami investigated in 13-year-old children was reported to vary according to their weight status, suggesting a relationship between umami sensitivity and children’s BMI [39]. To our knowledge, studies investigating suprathreshold taste responsiveness across five basic tastes in preadolescent children are still limited, as previous research mostly focused on preschoolers [40].

Understanding factors behind food choices and preferences in relation to taste responsiveness will help in developing effective intervention strategies to promote healthy eating in preadolescent children. This is especially relevant because childhood is a critical period for the development of obesity [41]. Moreover, this age group was reported to be at risk of becoming picky eaters [42,43]. A healthy food choice and eating behavior developed during childhood will remain until adulthood [44], so it is important to build healthy eating practices that can be pursued across the lifespan.

Individual variation in taste responsiveness can be investigated using taste stimuli diluted in water solutions [11,45,46] or in model foods with varying concentrations of taste compounds to alter the intensity of different target tastes [12,47,48]. Model foods were suggested for the study of taste sensitivity perception instead of water solutions since they are more representative of real food [49]. Responsiveness and preferences to sweet taste have been previously measured in children aged 5–10 years using model pudding varied with sucrose concentrations [48]. Other model foods such as crackers, broth, beverages, or soups, varied with different target taste compounds, have been previously used to study children’s taste sensitivity and preferences [12,13,16,50]. However, a study reported by Samant and Chapko [51] suggests that the use of a single tastant in water solution can minimize cross-modal interactions and/or product information effects.

The main objective of this study was to investigate the relationship between taste responsiveness and food liking in preadolescent children. Grapefruit juice and vegetable broth were used as model foods and four levels of tastant concentration were selected to induce a variation of basic tastes intensity for each series. Individual differences in the relationships between perceived taste intensity and liking in model foods were investigated, and four segments of children differing in basic taste responsiveness were identified. The relationships between children’s taste responsiveness across the different segments and PROP intensity perception, food choices, stated food liking, food familiarity, and food neophobia were also explored.

## 2. Materials and Methods

### 2.1. Participants

A total of 165 seventh-grade children were invited from three primary schools located in the Nordre Follo region, in Norway. A signed written consent from the children and their parents was required to participate in the study with one school providing the consent form digitally. A total of 148 children completed the tests (mean age = 11.9 ± 0.3 years, 48% boys). The school classes were rewarded for participating in the study, though each child’s participation was voluntary. Prior to the evaluation, we emphasized that the children could withdraw at any time without any consequences. The ethical approval of this study was granted by The Norwegian Center for Research Data (NSD) No. 715734 and refers to the Declaration of Helsinki of using human subjects, while data protection followed the General Data Protection Regulation (GDPR) [52].

### 2.2. Model Food Samples

Grapefruit juice (GF) (Cevita, Bama AS, Norway) and vegetable broth (VB) (Maggi, Nestle SA, Norway) were used as model food samples in this study. GF was selected due to the natural presence of bitterness and sourness in this product [53], which can be suppressed by the addition of sucrose [54]. VB was selected because it contains monosodium glutamate (MSG) that is perceived as umami and does not hold any meat ingredients that are avoided in some religions and personal diets. The addition of sodium chloride into the broth was aimed to elicit saltiness. Moreover, the model foods had to be easy to prepare, store, transport and serve. Four different concentrations of added sucrose (0, 40, 80, 160 g/L) were evaluated in GF and four different concentrations of added sodium chloride (0, 3, 6, 12 g/L) were evaluated in VB. The juice itself already contains natural sugars (mainly fructose) around 6.9 g/L while the broth contains around 10 g/L of salt at the base. Therefore, this resulted in a final concentration of sugar at around 6.9, 46.9, 86.9 and 166.9 g/L, respectively in GF, while salt content became 10, 13, 16 and 22 g/L in VB. However, for clarity, the concentrations in this paper were referred to the amount of tastant added into the model foods. Sweetness, bitterness, and sourness were investigated as target sensations in GF, while saltiness and umami were considered in VB. The amount of tastants to be added in each GF and VB to elicit different intensities of target tastes was selected based on a pretest with trained panelists at the University of Florence (*n* = 4) and Nofima (*n* = 11), and then with Norwegian children aged 10–13 years (*n* = 9).

Pre-weighed amounts of sucrose were added to the GF and stirred until completely dissolved. The GF mixture was then filtered using a sieve to remove the fruit pulp and stored in a closed container at 4 °C before being transferred into disposable cups. The VB was prepared by adding 14 g of vegetable broth powder into one liter of hot water (80 °C) and pre-weighted amounts of sodium chloride were added. The VB mixture was stirred until the broth powder and sodium chloride were completely dissolved, then filtered using a sieve to remove the small vegetable chunks. Excess fat formed at the surface of VB samples was removed using a spoon. All the food samples and taste compounds were food grade and purchased from a local supermarket. The sample preparation was conducted at the sensory laboratory at Nofima, Ås, and followed strict hygiene practices (i.e., using a mask, hand gloves, disinfecting the working surfaces, etc.).

The samples (20 mL) were served in 50 mL closed disposable cups and labeled with three-digit random codes. Each child received the samples in two boxes labeled as “box A” for liking evaluation and “box B” for taste responsiveness evaluation. Each box included four GF samples at different sucrose concentrations and four VB samples at different sodium chloride concentrations. Box A also included plain crackers for mouth rinsing (WASA, plain, gluten free and lactose free), while a PROP paper disc was provided in box B. In addition, water and spitting cups were also provided at the children’s tables. All samples were prepared one day before the evaluation, stored in a refrigerator at 4 °C overnight, and distributed to the school on the day of testing. The samples were kept at room temperature until the evaluation time, approximately 4 h from retrieval from the refrigerator.

### 2.3. Sensory Test Procedures: A Remote Testing Approach

**Figure 1:**
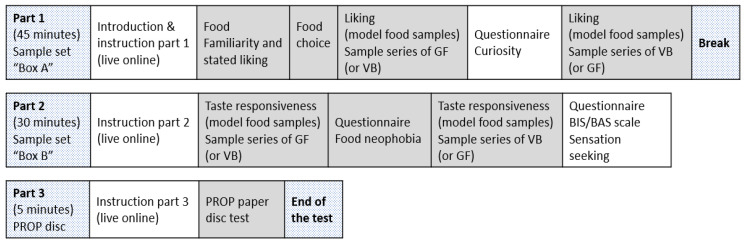
The study scheme (variables in grey show areas of interest reported in this paper).

The test was divided into three parts (Figure 1). In the first part, children filled in an online questionnaire on food familiarity, stated liking, and food choice of selected food items. In addition, liking data for model food samples were collected. In the second part, intensity perception responses on model foods were collected and children completed the food neophobia questionnaire. The last part aimed to measure children’s responsiveness to PROP bitterness on a paper disc. Note that the children also completed personality trait questionnaires and evaluated a list of food items and the model food samples for emotional responses; these results are not reported here.

All the tests were conducted at schools with one class taking the test at a time (15–22 participating children per class, 9 classes in total). Children were seated at individual tables, distanced from one another. The instructions were provided to the children at the beginning of each part (i.e., what the children should do, what samples they should taste, the explanation of the scales, etc.) with the support of a PowerPoint presentation (Microsoft Corporation, Redmond, Washington, United States). All instructions were provided via video conference call (Microsoft Teams, Microsoft Corporation, Redmond, Washington, United States) as there were restrictions in visiting the schools physically due to the Covid-19 pandemic. The video call was projected onto a large screen or smartboard in front of the class allowing the children to see and hear the instructions clearly. A video camera was turned on in the classroom during the entire evaluation, thus enabling the experimenters to monitor the test remotely. The children and teachers were able to ask questions directly to the instructor during the test and it took around two hours to finish the entire testing session (including a break). There was at least one teacher physically present in the room for the entire testing time, who assisted the experimenters with all practicalities in the classroom (i.e., placing the sample boxes on the children’s table, pouring the water for each child, helping with the camera and screen setting in the class, etc.). A separate discussion with the teachers took place before the evaluation day to inform them about the whole testing procedures, timing, and to ensure that good sensory practices would be followed during the test.

### 2.4. Food Familiarity and Stated Food Liking

Children rated familiarity and stated liking for 28 food items categorized as fruits (10), vegetables (10), juices and desserts (8) (Supplementary Materials Table S1). Vegetable items were selected among those regularly consumed by adolescents across Europe [55]. Fruits, juices, and desserts were selected to represent options differing in sweet, sour, and bitter intensity according to a previous study [56]. Food familiarity was evaluated on a five-point scale including 1 = “I do not know it”, 2 = “I know it, but I have never eaten this”, 3 = “I have tasted it, but I rarely eat it”, 4 = “I occasionally eat it”, and 5 = “I regularly eat it” [57]. Children who rated low familiarity with a given food item (1 = I don’t know; 2 = I know it, but I have never eaten this) were not asked to express their liking. Stated liking was measured on a seven-point hedonic scale ranging from “I dislike it very much” to “I like it very much”. The average scores for familiarity (1–5) and stated liking (1–7) were computed for each child based on their responses to the 28 food items. The food items were presented in a randomized order within and across categories.

### 2.5. Food Choice

A forced-choice method was applied to evaluate the children’s choice in 19 pairs of food items consisting of three categories of fruits (six pairs), vegetables (nine pairs), and juices and desserts (four pairs) (Supplementary Materials Table S2). The food items were paired within the same category, and they were selected to represent different intensities of bitter or sour tastes (lower vs. higher intensity) within the pair [58]. The vegetable pairs aimed to evaluate choice preference for bitter taste. For the selection of low/high bitter items in the vegetable pairs, data from a previous Check-All-That-Apply (CATA) questionnaire on 121 Italian preadolescents were used. This previous CATA questionnaire included a list of different vegetable names and four sensory descriptors: “sweet”, “sour”, “bitter”, and “delicate”. The six vegetable pairs in the present study were significantly different for bitterness citation frequency according to a Cochran’s Q test conducted on the CATA data: lettuce-rucola, spinach-lettuce, rucola-spinach, carrot-squash, squash-tomato, and broccoli-green beans (Supplementary Materials Table S3). In addition, differences in sweetness citation frequency were considered in three vegetable pairs: green beans-corn, green beans-carrots, and green beans-peas, assuming that vegetables with higher sweetness citation were less bitter. The fruits, juices, and desserts pairs aimed to evaluate choice preference for bitter and sour taste. The selection of items was based on a study by Martin et al. [56] who created a food taste database of multiple foods evaluated by a trained panel. For example, the pair of apple-orange represents different sourness intensities (less sour for apple and sourer for orange). The children’s task was to choose the food item that they preferred within the pair. The food pairs were evaluated in a randomized order within and across categories.

### 2.6. Model Food Evaluation (Liking and Taste Responsiveness)

Children’s liking for the model food samples was recorded using a Labeled Affective Magnitude Scale (LAM) [59,60]. The use of the scale was explained to the children prior to the evaluation. Moreover, examples of foods that are generally liked and disliked by children were recalled by name and picture (i.e., a slice of pizza vs. broccoli) and children were asked to express their liking on the LAM. This allowed the children to have a little training and practice on how to use the scale prior to the evaluation [61].

The children’s responsiveness to basic tastes in model food samples was recorded on the Labeled Magnitude Scale (LMS). The scale was labeled with intensity rating of barely detectable (1.4), weak (6.1), moderate (17.2), strong (35.4), very strong (53.3) and strongest imaginable (100) [62]. The five basic tastes qualities illustrated with pictures (i.e., sugar for sweetness, salt for saltiness, lemon for sourness, black coffee for bitterness, meat and soy sauce for umami) were recalled and explained to the children. The use of LMS was demonstrated to the children using pictures of foods with high and low intensity for the same taste quality (e.g., fresh lemon and lemonade for sourness, a spoon of salt and cheese for saltiness) [63]. The use of the scale was explained prior to the evaluation, and it was emphasized that there was no right or wrong answer in using the scale as it depends on one’s own perception.

To prevent positional bias, samples were evaluated in a randomized balanced order across and within GF and VB series across the children (Figure 1). During tasting, children were instructed to take a sip of the sample, swallow or expectorate the sample, and rate their liking (Part 1, Figure 1) or the intensity of target tastes (Part 2, Figure 1). The children were instructed to rinse their mouth with water in between tastings and to eat plain crackers to clean their palate. The tasting sessions were conducted autonomously and at individual speed by following the on-screen instructions. The break ensured that all children were ready for new common instructions at the start of Part 2, while waiting time could occur before the start of Part 3.

### 2.7. Food Neophobia

The children’s food neophobia was measured using the Italian Child Food Neophobia Scale (ICFNS), which consists of eight items (four neophobic and four neophilic statements) assessing the avoidance of trying new foods in children [64]. The scale was translated into Norwegian by a native speaker based upon its English version, then compared to the English version, the Swedish version and the original Italian version for adjustments [65]. The children’s responses were recorded using a five-point-agreement scale with anchors ‘‘very false’’, ‘‘false’’, ‘‘so-so’’, ‘‘true’’ and ‘‘very true’’ [64]. After reversal of the neophilic statements, the neophobia score was computed by summing up all the scores across statements for each child. Food neophobia scores ranged from 8 (low food neophobia) to 40 (high food neophobia). The Norwegian version of the scale is available in Supplementary materials.

### 2.8. PROP (6-n-prophylthiouracil)

The responsiveness to PROP was measured using the paper disc method [66,67] and the children’s responses were recorded using LMS [62]. The disc was impregnated with 50 mmol/L of PROP following a procedure from Zhao et al. [68]. Children were instructed to rinse their mouth with water before placing the PROP disc on the anterior part of their tongue (a picture with the correct position of the PROP disc on the tongue was presented to the children for guidance). Children were instructed to hold the PROP disc for 25 s in their mouth until it was completely soaked by their saliva, then take the paper out, wait for a further 20 s, and rate the bitterness that they perceived. The whole PROP disc testing process was individually guided with appropriate timers and instructions on screen. The test was allocated in the last part of the evaluation to refrain supertasters from being demotivated for further participation in the test. The PROP evaluation was performed 20 min after the model food tasting sessions to ensure that children did not have any lingering sensation from the previous samples.

### 2.9. Statistical Analysis

A mixed model ANOVA was applied to evaluate the effect of tastant concentration on the intensity of target sensations in model food samples. The statistical model was built separately for each taste (i.e., five models computed for sweetness, sourness, bitterness, saltiness, and umami) with taste intensity as the response variable, and concentrations (four concentrations of sucrose and sodium chloride in GF and VB, respectively) and gender as explanatory variables. The interaction between concentration and gender was also investigated, and child nested within gender was considered as a random effect (factors: concentration, gender (child), concentration × gender). The restricted maximum likelihood (REML) method was applied for fitting the model and post-hoc Tukey’s HSD test was computed.

A taste score was calculated for each child by summing up the intensity rated for each basic taste at the four concentration levels (e.g., taste score of sweet = sweet intensity at 0 + 40 + 80 + 160 g/L sucrose) [46]. A Principal Component Analysis (PCA) was then computed with children as rows and taste score of each taste as columns (five columns). The first two principal components were used to group the children into four different segments [69]. The PCA based segmentation was chosen because of good interpretability of the segments and more balance in cluster sizes which was important for subsequent statistical analysis (ANOVA). This approach is also referred to as interpretation-based on segmentation, and by this method the subjects can be split into segments based on primary interest [70]. Chi-square analysis was computed to check gender distribution across segments. The effect of segments, gender, and their interaction on taste score, PROP intensity, and mean liking was assessed by two-way ANOVAs (factors: segments, gender, segment × gender).

The effect of different segments and tastant concentrations (four levels) on taste intensity was computed per taste, using mixed model ANOVAs (five models were obtained). In these models, segment, concentration and interaction between concentration and segment were employed as explanatory variables, whereas child nested within segment was included as a random effect (factors: segment (child), concentration, segment × concentrations). The effect of segment and concentration on liking for model foods was also assessed using the same model and computed separately for GF and VB liking, respectively. Post hoc tests were performed using Tukey’s HSD test for pairwise comparison across concentrations within each segment.

A choice score was computed per child by summing up the total number of choices for the most sour and bitter options in each pair (choice score range: 0–19) [58]. The effect of segment and gender on food choice score was assessed using two-way ANOVA followed by post-hoc Tukey’s HSD test. A two-way ANOVA was also applied to evaluate the effect of segment and gender on children’s food neophobia, stated liking, and food familiarity (factors: segments, gender, segment × gender). In addition, further analyses for stated liking and familiarity as response variables were also computed using mixed model ANOVAs to investigate the effects of the different food items, segment, and gender (factors: segment, gender, food item, segment × gender, segment × food item, and food item × gender). Moreover, the correlation between children’s stated liking and familiarity was computed using Pearson correlation.

In all statistical tests, a threshold of 5% was applied to establish significance of an effect. All data analyses were computed using XLSTAT sensory version 2021.1.1 (Addinsoft, Paris France).

## 3. Results

### 3.1. Taste Intensity Perception in the Model Food Samples

The perceived intensity of sweetness, sourness, bitterness in GF, and saltiness in VB significantly changed according to the increase in tastant concentrations, while there were no significant changes observed for umami in VB (Table 1). Sweetness intensity significantly increased in parallel with the increase of sucrose concentration in GF, while intensity of sour and bitter tastes decreased. Saltiness intensity significantly increased in parallel with the increase of sodium chloride concentrations in VB, while umami taste intensity did not show a significant difference (*p* = 0.07). Gender did not significantly affect the intensity ratings of any of the basic tastes in the model food samples (*p* > 0.05).

### 3.2. Taste Responsiveness Segments

**Figure 2:**
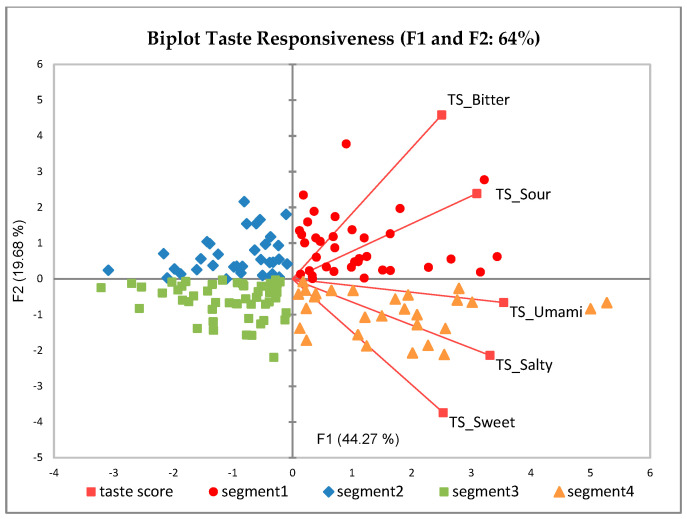
Children’s segmentation according to taste scores. Different colors and symbols indicate different segments (TS = Taste score).

The PCA bi-plot on taste responsiveness scores is reported in Figure 2. The first two principal components accounted for 64% of the total variability. The first principal component (44.3% of total variance) differentiates children into high responsive subjects on the right and low responsive subjects on the left side. The second principal component (19.7% of total variance) divided the children according to taste qualities, with children more responsive to generally well-liked tastes (sweet, salty, umami) on the bottom and those more responsive to generally disliked tastes (bitter and sour) on the top of the map. From the visual characterization of the map, four segments were identified with one segment for each quadrant in the PCA biplot [69].

According to the two-way ANOVA, each segment was significantly different for taste score (*p* < 0.001) and no gender difference was observed across segments (Table 2). Segment 1 (S1, *n* = 36, 24%) was characterized by the children who were highly responsive to bitterness and sourness compared to the other segments, and at the same time children in this segment were also less responsive to sweetness. Segment 2 (S2, *n* = 34, 23%) was characterized by the children who were least responsive to sweetness and moderately responsive to bitterness. Segment 3 (S3, *n* = 50, 34%) was characterized by the children who were low responsive to all basic tastes, and they were least responsive to bitter and sour compared to the other segments. Lastly, segment 4 (S4, *n* = 28, 19%) was mainly characterized by the children who were highly responsive to all basic tastes and have the highest responsiveness to sweet, salty and umami tastes across the segments. The intensity perception of PROP was significantly different across segments (*p* = 0.01) indicating that the children who were most responsive to PROP also had high taste responsiveness to all basic tastes (S4) or highly responsive to bitter and sour tastes (S1).

### 3.3. Segment Effect on Taste Intensity Perception in the Model Food Samples

**Figure 3:**
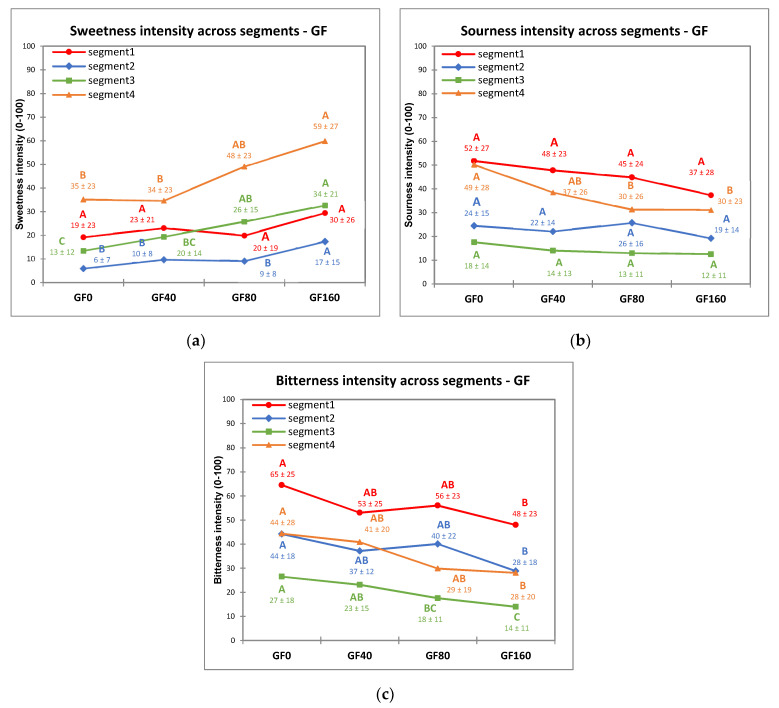
Taste intensity rating in grapefruit juice samples (mean intensity rating ± SD), (GF; 0–160 g/L added sucrose) for: sweetness (**a**), sourness (**b**) and bitterness (**c**) across the four segments. Different letters indicate significant differences (*p* < 0.05) from Tukey’s HSD test across concentrations within each segment.

The effect of segments and concentrations of sucrose (GF) or sodium chloride (VB) in model foods on perceived taste intensity was investigated separately for each basic taste using mixed model ANOVAs. The results demonstrate significant effects of segments (*p* < 0.001) and concentrations (sucrose/sodium chloride) for sweet (*p* < 0.001), sour (*p* < 0.001), bitter (*p* < 0.001), salty (*p* < 0.001), and umami (*p* = 0.03). The interactions between segments and concentrations were significant for sweet (*p* = 0.005) and sour (*p* = 0.022) tastes. The four segments showed differences in mean intensity values of target tastes based on taste scores (Table 2) and specific trends of intensity vs. tastant concentrations (Figure 3) in GF samples.

In S1, consisting of subjects highly responsive to bitterness and sourness and less responsive to sweetness, the increase of sucrose concentration in GF did not induce significant changes in neither sweetness nor sourness intensity, while only a weak but significant decrease of bitter intensity was observed. Sweetness was rated at moderate level in all samples for S1 while both bitterness and sourness were rated close to strong/very strong intensity. Thus, in this segment, sucrose addition did not significantly enhance sweetness nor suppress sourness intensity but only induced a weak suppression of bitterness. S3 was characterized by subjects with generally low responsive to all basic taste and the least responsive to both bitter and sour taste. In this segment, the increase of sucrose concentration induced a significant increase of sweetness intensity from weak to strong, associated to a significant suppression of bitterness from strong/moderate to weak, while sourness was rated as moderate/weak in all samples. S2 consisted of the children who were least responsive to sweetness. The intensity of sweetness in S2 changed from weak to weak/moderate with the increase of sucrose addition, while no significant changes were observed in sourness intensity that was rated moderate/strong in all samples, and a small but significant decrease of bitterness was observed in a range of strong/very strong intensity. Thus, in this segment, the increase of sucrose induced very small changes in sweetness, did not suppress sourness and slightly suppressed bitterness. S4 consisted of children that were highly responsive to all target tastes and showed the highest responsiveness to sweet taste; in this segment the increase of sucrose concentration induced a significant increase of sweetness intensity from strong to very strong level and a significant decrease of bitterness from very strong to strong, while sourness tended to decrease significantly at intermediate sucrose concentrations. Thus, in this segment a significant suppression of both bitterness and sourness was observed.

**Figure 4:**
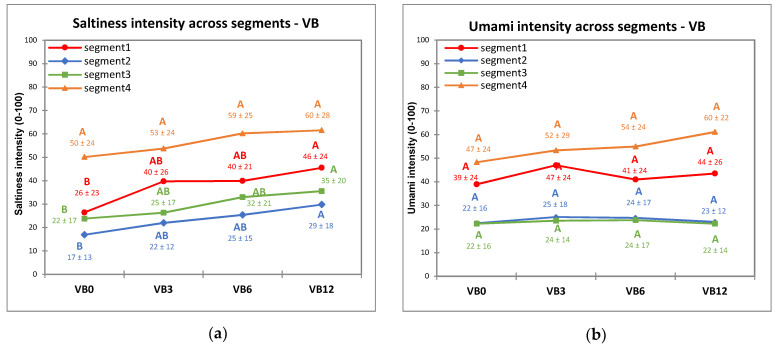
Taste intensity rating in vegetable broth samples (mean intensity rating ± SD), (VB; 0–12 g/L added sodium chloride) for: saltiness (**a**) and umami (**b**) across the four segments. Different letters indicate significant differences (*p* < 0.05) from Tukey’s HSD test across concentrations within each segment.

For the VB, segments S1, S2 and S3 had similar responses to saltiness with a significant increase in intensity response along with the increase of sodium chloride concentration, from moderate to strong in S2 and S3, and in strong/very strong range for S1, while S4 showed the same high saltiness intensity perception (very strong) in the whole sodium chloride concentrations range (Figure 4a). There were no differences for umami intensity responses across different salt concentrations in VB for any of the segments (Figure 4b). Umami intensity was of close to moderate intensity for S2 and S3, and ranged strong/very strong intensity for S1 and S4.

### 3.4. Taste Intensity Perception and Children’s Liking of Model Foods

There were significant differences in mean liking for GF and VB across segments (*p* < 0.001) (Table 2). The results demonstrated that children in S1, characterized by high responsiveness to sour and bitter tastes, and S2, low responsive to sweet taste and moderately responsive to bitter taste, had a significantly lower mean liking for GF compared to the other segments. Children in S3 with generally low responsiveness to basic tastes and with the lowest bitterness and sourness responsiveness had a higher mean liking for GF samples compared to S1 and S2. S4, which consisted of the children who were highly sensitive to all basic tastes and were the most responsive to sweet taste, showed the highest mean liking for GF samples. For VB, S1 showed the lowest mean liking score compared to the other segments while there were no differences between S2, S3, and S4 (Table 2).

**Figure 5:**
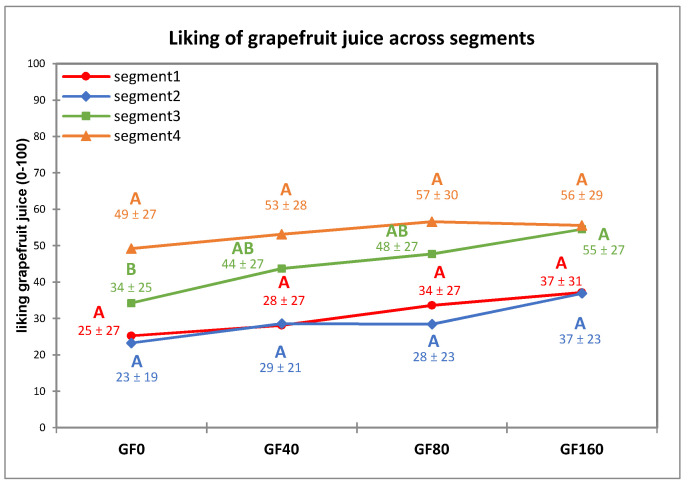
The effect of segment and sucrose concentration on liking for grapefruit juice (mean liking ± SD), (GF; 0–160 g/L added sucrose). Different letters indicate significant differences (*p* < 0.05) from Tukey’s HSD test across concentrations within each segment.

The differences among segments for liking in model foods was further investigated (Figure 5). There were significant effects of segment, concentration, and their interaction on the liking of GF (*p* < 0.001). Sucrose concentration positively affected liking in S3 only, showing a gradual increase of liking when the sucrose concentration is increased, while no significant changes in liking were found for other segments. There was no significant difference for liking score across the different sodium chloride concentrations in VB within each segment.

### 3.5. The Relationships between Taste Responsiveness Segments, Food Choice, Stated Food Liking, Familiarity, and Food Neophobia

In the choice task, children who were highly responsive to bitter and sour tastes (S1) and those who were least responsive to sweet and moderately responsive to bitter tastes (S2) tended to have a lower choice score for sour and/or bitter food options (*p* = 0.07) (Table 3). This result indicates that these segments (S1 and S2) tended to have lower preferences towards bitter and/or sour food. There was no significant effect of segments on the stated food liking. However, the different food items were rated differently by the children (*p* < 0.001) with milk chocolate being the most liked (6.6 ± 0.7) and green beans as the most disliked item (3.6 ± 1.1).

The segments did not differ in terms of food familiarity. However, the familiarity score was different across gender (*p* = 0.04), as girls had a slightly higher familiarity score compared to boys for seven of the items (milk chocolate, pineapple, grape, kiwi, green beans, fruit yogurt, and strawberry sorbet). The familiarity of the different food items was also shown to be significantly different (*p* < 0.001) with milk chocolate (4.3 ± 0.6) and apple (4.3 ± 0.7) having the highest familiarity score, while rucola (2.2 ± 1.2) and green beans (2.4 ± 0.9) were the least familiar. There was a significant positive correlation (r= 0.50, *p* < 0.001) between children’s stated liking and food familiarity.

The computed Cronbach’s alpha on the food neophobia measure was 0.80 showing good internal consistency of the questionnaire. Our data did not show a significant difference in food neophobia across segments (*p* = 0.27), indicating no systematic relationship between taste responsiveness scores and food neophobia. However, there was a gender effect (*p* = 0.01) indicating that boys were more neophobic compared to girls.

## 4. Discussion

### 4.1. Children’s Responsiveness to the Basic Tastes

The use of model food samples with varied concentrations of tastant (sucrose and sodium chloride) was shown to be effective in inducing different intensities of target taste sensations (sweetness and saltiness, respectively). Sucrose has been reported as a strong suppressor for bitter and sour taste [54]. The mean intensity perception of sweetness in GF gradually increased with sucrose concentration and at the same time both sourness and bitterness gradually decreased. Salty and umami tastes could enhance each other since these tastes work synergically [71,72]. However, umami intensity was not affected by the different concentrations of sodium chloride in VB samples in this study. This could be due to confusion of umami taste with saltiness or bitterness [73], since umami has been reported as the least familiar taste compared to other basic taste modalities in children aged 7–11 years [74].

Our subjects showed quite distinct differences in taste responsiveness for sweetness, sourness, bitterness, and saltiness (but not in umami) measured in the model food samples varying in sucrose (GF) or sodium chloride (VB) concentrations. It was thus possible to characterize the children into four segments with distinctive taste responsiveness profiles: high responsive to bitter and sour (S1), low responsive to sweet (S2), generally low responsive to all basic tastes with the lowest responsiveness to bitterness and sourness (S3), and generally high responsiveness to all basic tastes with the highest responsiveness to sweetness, saltiness, and umami (S4).

There were no significant differences for basic taste responsiveness across genders. This confirms previous work where no differences were found between boys and girls of a similar age group for their basic taste responsiveness measured in water solutions [11]. Moreover, PROP intensity was in accordance with the segments’ configuration, as the children who showed to be highly responsive to bitter and sour tastes (S1) and the children who were generally responsive to all basic tastes (S4) rated PROP intensity higher than the other two segments. These results further corroborate previous findings, as PROP intensity has previously been reported to be positively associated with the perceived intensity of basic tastes in children [8,11,75].

The suppression effect of sweetness (from sucrose) on bitterness and sourness intensity perception in GF was significantly related to the different taste responsiveness profiles of the four segments. In fact, sucrose addition in GF samples significantly suppressed sourness and bitterness intensity perception only in subjects with high responsiveness to sweetness (S4) and low responsiveness to sourness and bitterness (S3). On the other hand, a low responsiveness to sweetness (S2) or a high responsiveness to sourness and bitterness (S1) strongly lowered the sucrose suppression to bitterness and sourness intensity. Taste responsiveness also affected the discrimination ability of subjects among samples with increasing sucrose concentration in GF. S4 showed a sharp increase in perceived sweetness intensity at the highest sucrose concentrations (GF40-GF160, Figure 3a), this segment also significantly perceived decreased sourness and bitterness across GF samples in parallel with the increase of sucrose. This indicates that high-responsive children are more sensitive towards variations in tastant concentration [46,76]. Highly responsive subjects are able to perceive smaller variations of different tastant concentrations compared to less responsive subjects [46]. However, this phenomenon was not observed in VB samples for salty and umami tastes, as S4, which was the most responsive segment to these tastes, did not discriminate the different intensity levels among the samples. This indicates that different tastants and concentrations have different suppression and enhancement effects, and may influence taste intensity perception differently [72]. Another possibility could be that children may have already perceived a strong saltiness sensation in VB0 because the broth itself already contain salt (10 g/L), therefore further addition of salt in VB did not significantly increase saltiness perception. Moreover, children might also confuse the tastes of umami and salty [74] which may also influence the result in this study. In addition, the model food matrix of VB as “drink” samples and the fact that it was evaluated at room temperature may influence the intensity perception of children, as it is very uncommon to drink cold broth.

### 4.2. Relationships between Taste Responsiveness Segments and Liking of the Model Foods: Supression of Warning Sensation

Liking for GF samples was significantly different across segments but was not affected by sucrose concentration except for S3. Children in S1 and S2 were demonstrated to have a lower liking score for GF samples compared to the other segments. Sweetness suppression to warning sensations (bitterness and sourness) was probably not very effective due to high responsiveness to bitter and sour tastes in S1, and due to low responsiveness to sweet taste in S2. In addition, S2 was also moderately responsive to bitterness. In both segments (S1 and S2), the increase of sucrose concentration had no or very slight impact on sweetness intensity, and this was combined with a constant sourness intensity and only a slight decline in bitterness intensity. These results might explain the overall lower liking for GF observed in S1 and S2. Children in S4 were very responsive to sweetness, and this sensation was perceived as strong even at 0 g/L of sucrose addition (GF0) and increasingly high along with the increase of sucrose concentration. This possibly explains the same high liking score for GF samples regardless of the sucrose concentration in S4. In S3, sucrose addition significantly increased the intensity of sweetness and decreased bitterness intensity, thus explaining the significant increase of liking in GF across sucrose concentrations observed in this group. Subjects in S3 also had the lowest responsiveness for sour and bitter tastes. For these subjects, an effective suppression of the warning sensations of sourness and bitterness occurred by addition of sucrose.

S4 consisted of subjects with high taste responsiveness, and it is possible for these subjects to enjoy their foods at lower concentration of tastants and be satisfied at this level; their expectations may be met at lower levels of tastants compared to less sensitive subjects [77]. In contrast, subjects with low taste sensitivity will seek a higher degree of tastant concentration to meet their hedonic expectation [77,78]. This could be the reason why subjects in S3 liked the sweetest sample the most and kept increasing their liking with the increase of sucrose concentration. Indeed, S3 showed a generally low responsiveness to all basic tastes. In line with the previous literatures, our results further proved the strong association of sweetness with acceptance [18,79]. Furthermore, a previous study indicated that higher bitter sensitivity could hinder preferences toward bitter-sour drinks such as grapefruit juices in 9–11-year-old children [28]. This is in line with our results, since S1 and S2, both associated with highly responsive and moderately responsive subjects to bitter taste, respectively showed a lower overall liking score in GF.

### 4.3. Relationships between Taste Responsiveness Segments and Liking of the Model Foods: Role of Target-Taste Levels and Product Choice

Previous studies [80,81,82] classified three different groups of subjects according to their hedonic response to sweetness. The sweet likers group represented subjects who increase their liking as sweetness intensity increased (positive correlation). The inverted U-shape group is characterized by subjects who have a maximum liking for a certain sweetness intensity, whereas after this point their liking will decrease. The sweet dislikers group is characterized by subjects who decrease their liking when sweetness intensity is increased (negative correlation). According to our results, children in S3 (less responsive) could be categorized as sweet likers since their hedonic response increased significantly in parallel with the increase of sucrose concentration across the GF samples.

There were no significant effects of the taste responsiveness segments on the liking of VB samples. We assumed this was due to the strong saltiness intensity because the vegetable broth powder itself already contains sodium chloride as one of the ingredients at around 10 g/L. Moreover, in contrast to grapefruit juice, broth is not normally consumed by itself in a real-life situation. It is unusual to serve vegetable broth as a sample drink solely, therefore this may have led to unreliable hedonic responses despite clear differences in taste responsiveness. In association with the GF results, the VB results show that the relationship between taste responsiveness and liking is product and basic taste-dependent in addition to being subject-dependent.

### 4.4. Relationships between Taste Responsiveness Segments, Stated Liking and Familiarity

There was no significant pattern between the children’s segments on taste responsiveness and their stated liking of the selected food items. Children’s food liking is not solely affected by taste responsiveness; other extrinsic factors such as food exposure [83], parental modelling and feeding practices at home [84], and socio-demographic condition [85] were reported to be strongly associated with children’s food acceptance. Our results did not show any significant relationship between taste responsiveness and familiarity; this corroborates a previous study [58] that reports no association between bitter responsiveness of PROP and familiarity of vegetables differing in bitterness and astringency levels in an adult population. Furthermore, our data demonstrated a positive correlation between food familiarity and stated liking, indicating that the more often children are exposed to certain foods, the more they will become familiar with the foods, which could increase their acceptance [83,86,87,88].

### 4.5. Relationships between Taste Responsiveness Segments, Food Choice, and Food Neophobia

There were no significant differences in taste responsiveness segments in terms of food choice. However, there was a trend whereby the children who were responsive to bitterness and sourness (S1) and children who were less responsive to sweetness in addition to being moderately responsive to bitterness (S2) had a lower choice score for bitter/sour food option compared to the other segments. This indicates that these segments tended to not prefer bitter and/or sour food options. This result is in line with previous research which reported that adult subjects with high responsiveness to bitterness and sourness preferred foods that were less bitter and/or sour [89]. Moreover, bitter taste is strongly associated with food aversion [18] and children in S1 and S2 have higher responsiveness to bitter taste which makes them may avoid intense bitter foods. However, we have to consider that the selection of food items for the fruits, juices and desserts categories was based on sensory characterization reported by adult trained panelists [56] and not by preadolescent children. This could lead to a bias as children have a different taste intensity perception from adults [16,79,90,91]. In addition, the CATA-based sensory characterization of vegetables that was used for the selection of vegetable items was evaluated by Italian preadolescents, while the present study was conducted with Norwegian preadolescents. Cultural differences in sensory perception might occur [92] and influence the results in choice score preference since taste sensitivity in children aged 6–9 years has been reported to be significantly different across different countries [13].

The high internal consistency in the Norwegian version of the ICFNS (Cronbach’s alpha 0.8) was in line with previous validations of the scale in other languages [65]. Corroborating previous literature, we observed a tendency for boys to be more neophobic than girls [93,94]. However, it should be noted that no such gender effect was reported in a larger cross-cultural study also using the ICFNS [65]. Further, no systematic relationship between taste responsiveness scores and food neophobia occurred, indicating that a neophobic character trait poorly relates to taste perception ability. Similarly, Mameli et al. [95] reported that despite clear differences in taste recognition ability, fungiform papillae density, and responsiveness to PROP, no significant differences emerged in food neophobia scores between a group of Type 1 diabetics and a control group in children aged 6–15 years. Lafraire et al. [96] have highlighted the important role of cognitive, social, and environmental factors in food neophobia and picky/fussy eating behaviour.

### 4.6. Remote Sensory Testing

An original aspect of our study is that, due to the Covid-19 regulations, the sensory testing was conducted in schools with a teacher physically present in the classroom, while the experimenters interacted remotely via video conference call with the children. Some technical challenges need to be considered when running sensory testing remotely, such as the availability of devices (laptop or tablet) for each child, a large screen, camera, and speaker equipment in the classrooms to allow interaction with the experimenters, and a stable internet connection. In our case, the remote testing was technically easy to set up as each child was already equipped with a tablet or laptop provided by their schools. Moreover, many Norwegian preadolescents use their school tablet or laptop as their learning device at school as well as for homework on a daily basis, making them fully autonomous for the online set up and testing. In addition, the class setting was also equipped with a smartboard or smart screen, speaker, and school Wi-Fi which made the remote test possible.

After over a year of the Covid-19 pandemic, more preadolescents in Europe are expected to have received equipment and increased their digital literacy skills to adapt to online learning. This creates a new potential for application of remote sensory testing with preadolescents. Moreover, this method also allows recruitment of participants from other regions than where the experimenters’ working place is located, as long as the test samples can be delivered. Finally, remote testing is less invasive into the children’s comfort space; while physical testing in schools involves strangers (experimenters) invading the classroom, which may be stressful for timid children and exciting for extrovert children [97], remote testing keeps the experimenters on screen. Physical interactions only occur with familiar, safe adults from the school personnel. This may potentially reduce both stress and excitement among children, favoring a better focus on the task. Further studies are recommended to validate remote testing as an approach of data collection for this age group.

### 4.7. Implications for Strategy Development in Children’s Food Acceptance

Results of the present study indicate a prominent role of taste responsiveness on preadolescents’ acceptance of food characterized by warning sensations. Individual variations in responsiveness to both liked and disliked sensations not only modulates the perceived intensity but shapes taste interactions and hedonic responses. Our results indicate that strategies aimed at improving acceptance through the use of suppressors of generally disliked sensations can be more or less effective in subject groups varying in responsiveness to basic tastes and suggest the need for taking into account individual differences. For example, the food formulation strategies using cross-modal interactions (i.e., taste/texture/odor) could help to optimize food formulation [98] in order to overcome the low acceptance due to differences in taste responsiveness. Moreover, individual differences in taste responsiveness could modulate the effectiveness of masking strategies for tastes that generally have a low acceptance such as bitter taste. For example, masking bitterness with sugar (sweet) may be less effective to increase the acceptance of bitter vegetables in children characterized by high responsiveness to bitter taste, and thus other strategies such as repeated exposure may be suggested [86,88].

Increased awareness of the importance of individual perceptual differences in driving food preference and choice has been reported previously [47]. This calls for sensory-driven solutions in personalized nutrition recommendations to help vulnerable groups (i.e., obese children) to adopt a long-term healthy eating habit. Moreover, food preference is not shaped by taste sensitivity solely, but other extrinsic factors may strongly influence children’s food preferences [13,85]. This requires further research to explore a wider perspective on how taste responsiveness impacts both food preference and response to interventions, as well as investigating extrinsic factors related to food preferences in children. The implementation for “real-life” intervention cannot rely on sensory aspect only; however, a deeper understanding in sensory perceptions and hedonic responses to food might help to interpret food related behaviour and could effectively complement other actions aimed to improve healthy eating behaviour in children. Communicating these knowledges to professional and public bodies would allow the establishment of more effective healthy eating interventions which take into account the diversity of shaping food habits including individual differences in sensory perceptions.

### 4.8. Study Limitations

There were some limitations in conducting this study. First, we could not fully avoid interactions between children, as some of the classrooms were not large enough to arrange a satisfactory distance between peers. However, instructions on working individually during the test as well as supervision by the teacher and through video call ensured that interactions were kept to a minimum. Second, the food items selection for stated liking, familiarity, and food choice focused on different intensity levels for sweet, sour, and bitter but did not involve salty and umami foods. We suggest considering foods representing all basic tastes for further investigation. Third, model foods that are not normally consumed in ecological settings (such as room-temperate vegetable broth) do not seem to be appropriate test samples. Alternative food matrices should be identified for future studies on saltiness and umami. Lastly, the segments formed consisted of a low number of subjects; repeated studies and/or larger numbers of participants are suggested in future research to confirm the results obtained from this relatively small number of subjects.

## 5. Conclusions

This study aimed to investigate the relationships between taste responsiveness and liking in preadolescent children. Model food samples of grapefruit juice and vegetable broth with different concentrations of sucrose and sodium chloride, respectively, were employed to measure children’s perceived taste intensity and their liking. Four segments were formed according to children’s individual differences in taste responsiveness. The results showed that taste responsiveness significantly influenced the liking of grapefruit juice samples. However, children expressed little hedonic variations for the broth, despite clear significant variations in taste responsiveness for the same samples, indicating that the relationship between taste responsiveness and liking is product and target-taste dependent in addition to being subject-dependent.

This study also demonstrates that the suppression effect of sweetness on warning sensations of bitterness and sourness is associated with taste responsiveness in preadolescent children. Children who were highly responsive to bitterness and sourness and less responsive to sweetness did not experience a suppression effect of warning sensations by sweetness and this hindered the liking of model food sample of grapefruit juices. On the contrary, children who were least responsive to bitter and sour tastes showed increased liking as sucrose concentrations increased. This result calls for the development of different strategies specific to children’s taste responsiveness profiles, to increase their acceptance for foods dominated by warning sensations of sourness and bitterness such as fruits and vegetables. This study also confirmed a positive association between food familiarity and stated liking. A gender effect was observed for familiarity and neophobia, where boys were more neophobic and had lower familiarity scores compared to girls.

To our knowledge, this is the first study that employs a remote sensory evaluation method with preadolescent children to investigate their basic taste responsiveness and liking in model food samples. The usage of remote sensory testing as an alternative approach for sensory data collection in preadolescents is suggested for further study. Further research may investigate if the associations between taste responsiveness and liking are stable across different model food samples, basic tastes, and cultures.

## Figures and Tables

**Table 1:** Mean taste intensity ratings in model food samples with increasing tastant concentrations (sucrose in grapefruit juice, sodium chloride in vegetable broth).

Food Samples and Target Tastes	Sample 1 (Mean ± SD)	Sample 2 (Mean ± SD)	Sample 3 (Mean ± SD)	Sample 4 (Mean ± SD)	*p*-Value
**Grapefruit**	**GF 0 g/L**	**GF 40 g/L**	**GF 80 g/L**	**GF 160 g/L**	**across samples**
**juice (GF)**					
Sweetness	17.1 ± 20.0 ^c^	20.9 ± 18.2 ^bc^	24.9 ± 21.3 ^b^	33.6 ± 26.2 ^a^	F = 28.9, ***p* < 0.001**
Sourness	33.6 ± 26.0 ^a^	28.6 ± 23.1 ^b^	27.0 ± 22.5 ^bc^	23.5 ± 22.0 ^c^	F = 12.5, ***p* < 0.001**
Bitterness	43.2 ± 26.1 ^a^	36.9 ± 21.6 ^b^	34.4 ± 24.0 ^b^	28.3 ± 22.0 ^c^	F = 24.2, ***p* < 0.001**
**Vegetable**	**VB 0 g/L**	**VB 3 g/L**	**VB 6 g/L**	**VB 12 g/L**	**across samples**
**broth (VB)**					
Saltiness	27.2 ± 22.6 ^c^	33.2 ± 23.5 ^b^	37.6 ± 23.6 ^ab^	41.1 ± 25.0 ^a^	F = 23.6, ***p* < 0.001**
Umami	31.0 ± 22.4 ^a^	35.0 ± 24.6 ^a^	33.7 ± 23.7 ^a^	34.7 ± 24.4 ^a^	F = 2.4, *p* = 0.07

Different letters in rows indicate significant differences (*p* < 0.05) between mean values from Tukey’s HSD test. Values in bold show a significant difference at *p* < 0.05.

**Table 2:** Segment profiles according to taste score, perceived intensity, PROP intensity, and mean liking for model foods.

Variables	Segment 1 (S1) High Responsive to Bitter and Sour	Segment 2 (S2) Low Responsive to Sweet	Segment 3 (S3) Low Responsive to All Basic Tastes	Segment 4 (S4) High Responsive to All Basic Tastes	*p*-Value
All children					
(*n* = 148)	36 (24%)	34 (23%)	50 (34%)	28 (19%)	Chi-square,
Boys	18 (50%)	19 (56%)	18 (36%)	16 (57%)	gender
Girls	18 (50%)	15 (44%)	32 (64%)	12 (43%)	*p*= 0.19
Taste scores (0–400)					
Sweet (GF)	91.6 ^b^	41.7 ^c^	92.2 ^b^	175.9 ^a^	F = 36.4, ***p* < 0.001**
Sour (GF)	181.6 ^a^	91.3 ^b^	58.1 ^b^	146.7 ^a^	F = 35.0, ***p* < 0.001**
Bitter (GF)	221.7 ^a^	149.4 ^b^	81.5 ^c^	141.2 ^b^	F = 39.4, ***p* < 0.001**
Salty (VB)	151.6 ^b^	93.2 ^c^	114.7 ^bc^	222.3 ^a^	F = 23.0, ***p* < 0.001**
Umami (VB)	170.3 ^b^	93.9 ^c^	91.6 ^c^	213.5 ^a^	F = 29.6, ***p* < 0.001**
PROP mean intensity (LMS 0–100)	57.4 ± 28.1 ^a^	38.9 ± 28.6 ^b^	39.8 ± 28.0 ^b^	51.1 ± 30.0 ^ab^	F = 3.52, ***p* = 0.01** gender *p* = 0.32
Mean liking					
(LAM 0–100)					
GF (mean of 4 samples)	31.1 ± 28.0 ^c^	29.1 ± 22.3 ^c^	45.3 ± 27.1 ^b^	54.3 ± 29.0 ^a^	F = 75.5, ***p* < 0.001**
VB (mean of 4 samples)	30.0 ± 26.4 ^b^	40.6 ± 25.7 ^a^	37.5 ± 27.7 ^a^	37.3 ± 31.3 ^a^	F = 7.3, ***p* < 0.001**

Different letters in rows indicate significant differences (*p* < 0.05) between mean values from Tukey’s HSD test. Values in bold show a significant difference at *p* < 0.05. GF = Grapefruit juice, VB = Vegetable broth.

**Table 3:** Mean value for choice score, stated food liking, familiarity and neophobia according to the four taste responsiveness segments.

Variables	Segment 1 (S1) High Responsive to Bitter and Sour	Segment 2 (S2) Low Responsive to Sweet	Segment 3 (S3) Low Responsive to All Basic Tastes	Segment 4 (S4) High Responsive to All Basic Tastes	*p*-Value
Choice score (0–19)	5.5 ± 2.5 ^a^	5.5 ± 2.0 ^a^	6.4 ± 1.9 ^a^	6.6 ± 2.4 ^a^	F = 2.6, *p* = 0.07 gender *p* = 0.55
Stated food liking (1–7)	5.2 ± 0.5 ^a^	5.1 ± 0.6 ^a^	5.3 ± 0.5 ^a^	5.2 ± 0.5 ^a^	*p* = 0.81 gender *p* = 0.15
Food familiarity (1–5)	3.4 ± 0.5 ^a^	3.4 ± 0.4 ^a^	3.5 ± 0.3 ^a^	3.4 ± 0.5 ^a^	*p* = 0.43 gender ***p* = 0.04**
Food neophobia (8–40)	21.7 ± 6.2 ^a^	21.7 ± 5.1 ^a^	22.1 ± 6.3 ^a^	19.5 ± 6.4 ^a^	F = 1.2, *p* = 0.27 gender ***p* = 0.01**

Different letters in rows indicate significant differences (*p* < 0.05) from Tukey’s HSD test. Values in bold show a significant difference at *p* < 0.05.

## Data Availability

The data presented in this study are available at doi:10.5281/zenodo.5167132.

## Supplementary Materials

The following are available online at https://www.mdpi.com/article/10.3390/nu13082721/s1, Table S1: Selected food items for familiarity and stated liking, Table S2: Selected food items for food choice preference, Table S3: Statistical results (Cochran’s Q test) from a sensory characterization of vegetables with Check-All-That-Apply (CATA) in Italian preadolescent children (*n* = 121), Table S4: Norwegian version of the Italian Child Food Neophobia Scale (ICFNS).

## References

[1] Oellingrath I.M., Hersleth M., Svendsen M.V. (2013). Association between parental motives for food choice and eating patterns of 12- to 13-year-old Norwegian children. Public Health Nutr..

[2] Nguyen S.P., Girgis H., Robinson J. (2015). Predictors of children’s food selection: The role of children’s perceptions of the health and taste of foods. Food Qual. Prefer..

[3] Schwartz C., Chabanet C., Lange C., Issanchou S., Nicklaus S. (2011). The role of taste in food acceptance at the beginning of complementary feeding. Physiol. Behav..

[4] Schwartz C., Chabanet C., Szleper E., Feyen V., Issanchou S., Nicklaus S. (2017). Infant acceptance of primary tastes and fat emulsion: Developmental changes and links with maternal and infant characteristics. Chem. Senses.

[5] Boesveldt S., Bobowski N., McCrickerd K., Maître I., Sulmont-Rossé C., Forde C.G. (2018). The changing role of the senses in food choice and food intake across the lifespan. Food Qual. Prefer..

[6] De Cosmi V., Scaglioni S., Agostoni C. (2017). Early Taste Experiences and Later Food Choices. Nutrients.

[7] Drewnoski A. (1997). Taste preferences and food intake. Annu. Rev. Nutr..

[8] Keller K.L., Adise S. (2016). Variation in the Ability to Taste Bitter Thiourea Compounds: Implications for Food Acceptance, Dietary Intake, and Obesity Risk in Children. Annu. Rev. Nutr..

[9] Reed D.R., Knaapila A. (2010). Genetics of taste and smell: Poisons and pleasures. Prog. Mol. Biol. Transl. Sci..

[10] Sick J., Hojer R., Olsen A. (2019). Children’s Self-Reported Reasons for Accepting and Rejecting Foods. Nutrients.

[11] Ervina E., Berget I., Almli V.L. (2020). Investigating the Relationships between Basic Tastes Sensitivities, Fattiness Sensitivity, and Food Liking in 11-Year-Old Children. Foods.

[12] Alexy U., Schaefer A., Sailer O., Busch-Stockfisch M., Huthmacher S., Kunert J., Kersting M. (2011). Sensory Preferences and Discrimination Ability of Children in Relation to Their Body Weight Status. J. Sens. Stud..

[13] Ahrens W. (2015). Sensory taste preferences and taste sensitivity and the association of unhealthy food patterns with overweight and obesity in primary school children in Europe—A synthesis of data from the IDEFICS study. Flavour.

[14] Leonie B.H., Wolters M., Börnhorst C., Intemann T., Reisch L.A., Ahrens W., Hebestreit A. (2018). Dietary habits and obesity in European children. Ernaehrungs Umsch. Int..

[15] Lanfer A., Knof K., Barba G., Veidebaum T., Papoutsou S., de Henauw S., Soos T., Moreno L.A., Ahrens W., Lissner L. (2012). Taste preferences in association with dietary habits and weight status in European children: Results from the IDEFICS study. Int. J. Obes. (Lond.).

[16] Mennella J.A., Finkbeiner S., Lipchock S.V., Hwang L.D., Reed D.R. (2014). Preferences for salty and sweet tastes are elevated and related to each other during childhood. PLoS ONE.

[17] Vennerød F.F.F., Nicklaus S., Lien N., Almli V.L. (2018). The development of basic taste sensitivity and preferences in children. Appetite.

[18] Mennella J.A., Bobowski N.K. (2015). The sweetness and bitterness of childhood: Insights from basic research on taste preferences. Physiol. Behav..

[19] Glendinning J.I. (1994). Is the bitter rejection response always adaptive?. Physiol. Behav..

[20] Mennella J.A., Spector A.C., Reed D.R., Coldwell S.E. (2013). The bad taste of medicines: Overview of basic research on bitter taste. Clin. Ther..

[21] Kildegaard H., Tønning E., Thybo A.K. (2011). Preference, liking and wanting for beverages in children aged 9–14 years: Role of sourness perception, chemical composition and background variables. Food Qual. Prefer..

[22] Drewnowski A., Mennella J.A., Johnson S.L., Bellisle F. (2012). Sweetness and food preference. J. Nutr..

[23] van Stokkom V.L., Poelman A.A.M., de Graaf C., van Kooten O., Stieger M. (2018). Sweetness but not sourness enhancement increases acceptance of cucumber and green capsicum purees in children. Appetite.

[24] Chamoun E., Liu A.A.S., Duizer L.M., Darlington G., Duncan A.M., Haines J., Ma D.W.L. (2019). Taste Sensitivity and Taste Preference Measures Are Correlated in Healthy Young Adults. Chem. Senses.

[25] Garcia-Bailo B., Toguri C., Eny K.M., El-Sohemy A. (2009). Genetic Variation in Taste and Its Influence on Food Selection. OMICS A J. Integr. Biol..

[26] Puputti S., Aisala H., Hoppu U., Sandell M. (2019). Factors explaining individual differences in taste sensitivity and taste modality recognition among Finnish adults. J. Sens. Stud..

[27] Dinnella C., Monteleone E., Piochi M., Spinelli S., Prescott J., Pierguidi L., Gasperi F., Laureati M., Pagliarini E., Predieri S. (2018). Individual Variation in PROP Status, Fungiform Papillae Density, and Responsiveness to Taste Stimuli in a Large Population Sample. Chem. Senses.

[28] Hartvig D., Hausner H., Wendin K., Bredie W.L. (2014). Quinine sensitivity influences the acceptance of sea-buckthorn and grapefruit juices in 9- to 11-year-old children. Appetite.

[29] Joseph P.V., Reed D.R., Mennella J.A. (2016). Individual differences among children in sucrose detection thresholds: Relationship with age, gender, and bitter taste genotype. Nurs. Res..

[30] Prescott J., Tepper B.J. (2004). Genetic Variation in Taste Sensitivity.

[31] Drayna D. (2005). Human taste genetics. Annu. Rev. Genomics Hum. Genet..

[32] Dioszegi J., Llanaj E., Adany R. (2019). Genetic Background of Taste Perception, Taste Preferences, and Its Nutritional Implications: A Systematic Review. Front. Genet..

[33] Tepper B.J., Melis M., Koelliker Y., Gasparini P., Ahijevych K.L., Tomassini Barbarossa I. (2017). Factors Influencing the Phenotypic Characterization of the Oral Marker, PROP. Nutrients.

[34] Fischer M.E., Cruickshanks K.J., Pankow J.S., Pankratz N., Schubert C.R., Huang G.H., Klein B.E., Klein R., Pinto A. (2014). The associations between 6-n-propylthiouracil (PROP) intensity and taste intensities differ by TAS2R38 haplotype. J. Nutr. Nutr..

[35] Bell K.I., Tepper B.J. (2006). Short-term vegetable intake by young children classified by 6-n-propylthoiuracil bitter-taste phenotype. Am. J. Clin. Nutr..

[36] Stoner L., Castro N., Kucharska-Newton A., Smith-Ryan A.E., Lark S., Williams M.A., Faulkner J., Skidmore P. (2019). Food Consumption Patterns and Body Composition in Children: Moderating Effects of Prop Taster Status. Nutrients.

[37] Liem D.G. (2017). Infants’ and children’s salt taste perception and liking: A review. Nutrients.

[38] Kim G.H., Lee H.M. (2009). Frequent consumption of certain fast foods may be associated with an enhanced preference for salt taste. J. Hum. Nutr. Diet..

[39] Overberg J., Hummel T., Krude H., Wiegand S. (2012). Differences in taste sensitivity between obese and non-obese children and adolescents. Arch. Dis. Child..

[40] Nicklaus S. (2020). Eating and Drinking in Childhood. Handbook of Eating and Drinking.

[41] Cornwell T.B., McAlister A.R. (2011). Alternative thinking about starting points of obesity. Development of child taste preferences. Appetite.

[42] Houldcroft L., Farrow C., Haycraft E. (2014). Perceptions of parental pressure to eat and eating behaviours in preadolescents: The mediating role of anxiety. Appetite.

[43] Viljakainen H.T., Figueiredo R.A.O., Rounge T.B., Weiderpass E. (2019). Picky eating—A risk factor for underweight in Finnish preadolescents. Appetite.

[44] Nicklaus S., Remy E. (2013). Early Origins of Overeating: Tracking Between Early Food Habits and Later Eating Patterns. Curr. Obes. Rep..

[45] Puputti S., Aisala H., Hoppu U., Sandell M. (2018). Multidimensional measurement of individual differences in taste perception. Food Qual. Prefer..

[46] Piochi M., Dinnella C., Spinelli S., Monteleone E., Torri L. (2021). Individual differences in responsiveness to oral sensations and odours with chemesthetic activity: Relationships between sensory modalities and impact on the hedonic response. Food Qual. Prefer..

[47] Monteleone E., Spinelli S., Dinnella C., Endrizzi I., Laureati M., Pagliarini E., Sinesio F., Gasperi F., Torri L., Aprea E. (2017). Exploring influences on food choice in a large population sample: The Italian Taste project. Food Qual. Prefer..

[48] Mennella J.A., Finkbeiner S., Reed D.R. (2012). The proof is in the pudding: Children prefer lower fat but higher sugar than do mothers. Int. J. Obes. (Lond.).

[49] Dea S., Plotto A., Manthey J.A., Raithore S., Irey M., Baldwin E. (2013). Interactions and Thresholds of Limonin and Nomilin in Bitterness Perception in Orange Juice and Other Matrices. J. Sens. Stud..

[50] Liem D.G., Westerbeek A., Wolterink S., Kok F.J., de Graaf C. (2004). Sour taste preferences of children relate to preference for novel and intense stimuli. Chem. Senses.

[51] Samant S.S., Chapko M.J., Seo H.S. (2017). Predicting consumer liking and preference based on emotional responses and sensory perception: A study with basic taste solutions. Food Res. Int..

[52] European Parliament and Council of the European Union (2016). Regulation (EU) 2016/679 of the European Parliament and of the Council of 27 April 2016 on the Protection of Natural Persons with Regard to the Processing of Personal Data and on the Free Movement of Such Data, and Repealing Directive 95/46/EC (General Data Protection Regulation).

[53] Gous A.G.S., Almli V.L., Coetzee V., de Kock H.L. (2019). Effects of Varying the Color, Aroma, Bitter, and Sweet Levels of a Grapefruit-Like Model Beverage on the Sensory Properties and Liking of the Consumer. Nutrients.

[54] Green B.G., Lim J., Osterhoff F., Blacher K., Nachtigal D. (2010). Taste mixture interactions: Suppression, additivity, and the predominance of sweetness. Physiol. Behav..

[55] Dinnella C., Morizet D., Masi C., Cliceri D., Depezay L., Appleton K.M., Giboreau A., Perez-Cueto F.J.A., Hartwell H., Monteleone E. (2016). Sensory determinants of stated liking for vegetable names and actual liking for canned vegetables: A cross-country study among European adolescents. Appetite.

[56] Martin C., Visalli M., Lange C., Schlich P., Issanchou S. (2014). Creation of a food taste database using an in-home “taste” profile method. Food Qual. Prefer..

[57] Tuorila H., Lahtenmaki L., Pohjalainen L., Lotti L. (2001). Food neophobia among the Finns and related responses to familiar and unfamiliar foods. Food Qual. Prefer..

[58] De Toffoli A., Spinelli S., Monteleone E., Arena E., Di Monaco R., Endrizzi I., Gallina Toschi T., Laureati M., Napolitano F., Torri L. (2019). Influences of Psychological Traits and PROP Taster Status on Familiarity with and Choice of Phenol-Rich Foods and Beverages. Nutrients.

[59] Cardello A.V., Schutz H.G. (2004). Numerical Scale-Point Locations For Constructing The LAM (Labeled Affective Magnitude) Scale. J. Sens. Stud..

[60] Schutz H.G., Cardello A.V. (2001). A Labeled Affective Magnitude (LAM) Scale for Assessing Food Liking/Disliking. J. Sens. Stud..

[61] Lawless H.T., Popper R., Kroll B.J. (2010). A comparison of the labeled magnitude (LAM) scale, an 11-point category scale and the traditional 9-point hedonic scale. Food Qual. Prefer..

[62] Green B.G., Dalton P., Cowart B., Shaffer G., Rankin K., Higgins J. (1996). Evaluating the ‘Labeled Magnitude Scale’ for Measuring Sensations of Taste and Smell. Chem. Senses.

[63] Goldstein G.L., Daun H., Tepper B.J. (2007). Influence of PROP taster status and maternal variables on energy intake and body weight of pre-adolescents. Physiol. Behav..

[64] Laureati M., Bergamaschi V., Pagliarini E. (2015). Assessing childhood food neophobia: Validation of a scale in Italian primary school children. Food Qual. Prefer..

[65] Proserpio C., Almli V.L., Sandvik P., Sandell M., Methven L., Wallner M., Jilani H., Zeinstra G.G., Alfaro B., Laureati M. (2020). Cross-national differences in child food neophobia: A comparison of five European countries. Food Qual. Prefer..

[66] Oftedal K.N., Tepper B.J. (2013). Influence of the PROP bitter taste phenotype and eating attitudes on energy intake and weight status in pre-adolescents: A 6-year follow-up study. Physiol. Behav..

[67] Pickering G.J., Simunkova K., DiBattista D. (2004). Intensity of taste and astringency sensations elicited by red wines is associated with sensitivity to PROP (6-n-propylthiouracil). Food Qual. Prefer..

[68] Zhao L., Kirkmeyer S.V., Tepper B.J. (2003). A paper screening test to assess genetic taste sensitivity to 6-n-propylthiouracil. Physiol. Behav..

[69] Endrizzi I., Gasperi F., Rødbotten M., Næs T. (2014). Interpretation, validation and segmentation of preference mapping models. Food Qual. Prefer..

[70] Næs T., Varela P., Berget I. (2018). Individual Differences in Sensory and Consumer Science: Experimentation, Analysis and Interpretation.

[71] Hartley I.E., Liem D.G., Keast R. (2019). Umami as an ‘Alimentary’ Taste. A New Perspective on Taste Classification. Nutrients.

[72] Keast R.S.J., Breslin P.A.S. (2003). An overview of binary taste–taste interactions. Food Qual. Prefer..

[73] Melis M., Tomassini Barbarossa I. (2017). Taste Perception of Sweet, Sour, Salty, Bitter, and Umami and Changes Due to l-Arginine Supplementation, as a Function of Genetic Ability to Taste 6-n-Propylthiouracil. Nutrients.

[74] Mustonen S., Rantanen R., Tuorila H. (2009). Effect of sensory education on school children’s food perception: A 2-year follow-up study. Food Qual. Prefer..

[75] Feeney E.L., O’Brien S.A., Scannell A.G.M., Markey A., Gibney E.R. (2014). Genetic and environmental influences on liking and reported intakes of vegetables in Irish children. Food Qual. Prefer..

[76] Prescott J., Soo J., Campbell H., Roberts C. (2004). Responses of PROP taster groups to variations in sensory qualities within foods and beverages. Physiol. Behav..

[77] Cox D.N., Hendrie G.A., Carty D. (2016). Sensitivity, hedonics and preferences for basic tastes and fat amongst adults and children of differing weight status: A comprehensive review. Food Qual. Prefer..

[78] Papantoni A., Shearrer G.E., Sadler J.R., Stice E., Burger K.S. (2021). Longitudinal Associations Between Taste Sensitivity, Taste Liking, Dietary Intake and BMI in Adolescents. Front. Psychol..

[79] Forestell A.C., Mennella J.A., Doty R.L. (2015). The ontology of taste perception and preference throughout childhood. Handbook of Olfaction and Gustation.

[80] Iatridi V., Hayes J.E., Yeomans M.R. (2019). Quantifying Sweet Taste Liker Phenotypes: Time for Some Consistency in the Classification Criteria. Nutrients.

[81] Garneau N.L., Nuessle T.M., Mendelsberg B.J., Shepard S., Tucker R.M. (2018). Sweet liker status in children and adults: Consequences for beverage intake in adults. Food Qual. Prefer..

[82] Methven L., Xiao C., Cai M., Prescott J. (2016). Rejection thresholds (RjT) of sweet likers and dislikers. Food Qual. Prefer..

[83] Nicklaus S. (2016). Relationships between early flavor exposure, and food acceptability and neophobia. Flavor.

[84] Blissett J., Fogel A. (2013). Intrinsic and extrinsic influences on children’s acceptance of new foods. Physiol. Behav..

[85] Jilani H., Intemann T., Bogl L.H., Eiben G., Molnar D., Moreno L.A., Pala V., Russo P., Siani A., Solea A. (2017). Familial aggregation and socio-demographic correlates of taste preferences in European children. BMC Nutr..

[86] Cooke L. (2007). The importance of exposure for healthy eating in childhood: A review. J. Hum. Nutr. Diet..

[87] Aldridge V., Dovey T., Halford J. (2009). The Role of Familiarity in Dietary Development. Dev. Rev..

[88] Mohd Nor N.D., Houston-Price C., Harvey K., Methven L. (2021). The effects of taste sensitivity and repeated taste exposure on children’s intake and liking of turnip (Brassica rapa subsp. rapa); a bitter Brassica vegetable. Appetite.

[89] Pagliarini E., Proserpio C., Spinelli S., Lavelli V., Laureati M., Arena E., Di Monaco R., Menghi L., Gallina Toschi T., Braghieri A. (2021). The role of sour and bitter perception in liking, familiarity and choice for phenol-rich plant-based foods. Food Qual. Prefer..

[90] James C.E., Laing D.G., Oram N. (1997). A comparison of the ability of 8–9-year-old children and adults to detect taste stimuli. Physiol. Behav..

[91] De Graaf C., Zandstra E.H. (1999). Sweetness intensity and pleasantness in children, adolescents, and adults. Physiol. Behav..

[92] Cecchini M.P., Knaapila A., Hoffmann E., Boschi F., Hummel T., Iannilli E. (2019). A cross-cultural survey of umami familiarity in European countries. Food Qual. Prefer..

[93] Guzek D., Glabska D., Lange E., Jezewska-Zychowicz M. (2017). A Polish Study on the Influence of Food Neophobia in Children (10-12 Years Old) on the Intake of Vegetables and Fruits. Nutrients.

[94] Ulla-Kaisa K.H., Sjoden P.-O. (1997). Food and General Neophobia and their Relationship with Self-Reported Food Choice: Familial Resemblance in Swedish Families with Children of Ages 7–17 Years. Appetite.

[95] Mameli C., Cattaneo C., Lonoce L., Bedogni G., Redaelli F.C., Macedoni M., Zuccotti G., Pagliarini E. (2019). Associations Among Taste Perception, Food Neophobia and Preferences in Type 1 Diabetes Children and Adolescents: A Cross-Sectional Study. Nutrients.

[96] Lafraire J., Rioux C., Giboreau A., Picard D. (2016). Food rejections in children: Cognitive and social/environmental factors involved in food neophobia and picky/fussy eating behavior. Appetite.

[97] Jilani H., Peplies J., Buchecker K. (2019). Assessment of Sensory Taste Perception in Children. Instruments for Health Surveys in Children and Adolescents.

[98] Velazquez A.L., Vidal L., Varela P., Ares G. (2020). Cross-modal interactions as a strategy for sugar reduction in products targeted at children: Case study with vanilla milk desserts. Food Res. Int..

