# Supplementary material for: Does Responsiveness to Basic Tastes Influence Preadolescents’ Food Liking? Investigating Taste Responsiveness Segment on Bitter-Sour-Sweet and Salty-Umami Model Food Samples
Source: Nutrients. 2021 Aug 7;13(8):2721. doi: 10.3390/nu13082721 (PMC8401806; doi:10.3390/nu13082721)
Supplement: Supplementary file 1 [file nutrients-13-02721-s001.zip › S1 Supplementary Table 1.pdf]

Supplementary Table 1. Selected food items for familiarity and stated liking. The food item presented in a randomized order across subjects.

No	Food items
1	Apple
2	Clementine
3	Pineapple
4	Watermelon
5	Strawberries
6	Grapes
7	Grapefruits
8	Raspberries
9	Kiwi
10	Orange
11	Rucola
12	Spinach
13	Lettuce
14	Broccoli
15	Green beans
16	Squash
17	Peas
18	Tomato
19	Corn
20	Carrots
21	Orange juice
22	Grapefruit juice
23	Milk chocolate
24	Dark chocolate
25	Vanilla ice cream
26	Fruit yogurt
27	Plain yogurt
28	Strawberry sorbet
