# Supplementary material for: Does Responsiveness to Basic Tastes Influence Preadolescents’ Food Liking? Investigating Taste Responsiveness Segment on Bitter-Sour-Sweet and Salty-Umami Model Food Samples
Source: Nutrients. 2021 Aug 7;13(8):2721. doi: 10.3390/nu13082721 (PMC8401806; doi:10.3390/nu13082721)
Supplement: Supplementary file 1 [file nutrients-13-02721-s001.zip › S2 Supplementary Table 2.pdf]

Supplementary Table 2. Selected food items for food choice preference. Pairs were presented in a randomized order across subjects. (L) and (R) indicate a left or right position of the item in the questionnaire.

Pair No.	Category	Taste	Low taste intensity food	High taste intensity food
1	Fruit	Sour	Apple (L)	Orange (R)
2	Fruit	Sour	Watermelon (R)	Orange (L)
3	Fruit	Sour	Pineapple (R)	Grapefruit (L)
4	Fruit	Sour	Grapes (L)	Kiwi (R)
5	Fruit	Sour	Clementine (L)	Kiwi (R)
6	Fruit	Sour	Strawberries (L)	Raspberries (R)
7	Vegetable	Bitter	Lettuce (L)	Rucola (R)
8	Vegetable	Bitter	Lettuce (R)	Spinach (L)
9	Vegetable	Bitter	Spinach (R)	Rucola (L)
10	Vegetable	Bitter	Corn (R)	Green beans (L)
11	Vegetable	Bitter	Carrots (L)	Squash (R)
12	Vegetable	Bitter	Tomato (R)	Squash (L)
13	Vegetable	Bitter	Green beans (R)	Broccoli (L)
14	Vegetable	Bitter	Carrots (L)	Green beans (R)
15	Vegetable	Bitter	Peas (R)	Green beans (L)
16	Juice	Sour	Apple juice (R)	Grapefruit juice (L)
17	Dessert	Bitter	Milk chocolate (L)	Dark chocolate (R)
18	Dessert	Sour	Vanilla ice cream (L)	Lemon sorbet (R)
19	Dessert	Sour	Fruit yogurt (R)	Plain yogurt (L)
