# Supplementary material for: Does Responsiveness to Basic Tastes Influence Preadolescents’ Food Liking? Investigating Taste Responsiveness Segment on Bitter-Sour-Sweet and Salty-Umami Model Food Samples
Source: Nutrients. 2021 Aug 7;13(8):2721. doi: 10.3390/nu13082721 (PMC8401806; doi:10.3390/nu13082721)
Supplement: Supplementary file 1 [file nutrients-13-02721-s001.zip › S3 Supplementary Table 3.pdf]

Supplementary Table 3: Statistical results (Cochran's Q test) from a sensory characterization of vegetables with Check-All-That-Apply (CATA) in Italian preadolescent children (n=121)

Attributes	p-values	Lettuce	Rucola
Sour	**0.020**	0.038 (a)	0.124(b)
Delicate	**0.006**	0.343 (b)	0.190 (a)
Sweet	**0.008**	0.219 (b)	0.086 (a)
Bitter	**<0.001**	0.105 (a)	0.381 (b)

Attributes	p-values	Spinach	Lettuce
Sour	**0.033**	0.034 (a)	0.101 (b)
Delicate	**<0.001**	0.311 (b)	0.050 (a)
Sweet	0.106	0.218 (a)	0.143 (a)
Bitter	**0.047**	0.176 (b)	0.101 (a)

Attributes	p-values	Rucola	Spinach
Sour	0.819	0.124 (a)	0.114 (a)
Delicate	**0.002**	0.190 (b)	0.048 (a)
Sweet	0.108	0.086 (a)	0.152 (a)
Bitter	**<0.001**	0.381 (b)	0.152 (a)

Attributes	p-values	Green beans	Corn
Sour	0.206	0.025 (a)	0.058 (a)
Delicate	**<0.001**	0.042 (a)	0.333 (b)
Sweet	**<0.001**	0.208 (a)	0.417 (b)
Bitter	0.564	0.067 (a)	0.050 (a)

Attributes	p-values	Carrots	Squash
Sour	0.132	0.034 (a)	0.076 (a)
Delicate	**<0.001**	0.568 (b)	0.203 (a)
Sweet	**0.002**	0.356 (b)	0.178 (a)
Bitter	**0.007**	0.034 (a)	0.136 (b)

Attributes	p-values	Squash	Tomato
Sour	0.071	0.076 (a)	0.136 (a)
Delicate	**<0.001**	0.203 (b)	0.059 (a)
Sweet	**0.001**	0.178 (a)	0.356 (b)
Bitter	**0.003**	0.136 (b)	0.025 (a)

Attributes	p-values	Broccoli	Green beans
Sour	0.132	0.066 (a)	0.025 (a)
Delicate	**0.018**	0.124 (b)	0.041 (a)
Sweet	0.178	0.149 (a)	0.207 (a)
Bitter	**0.001**	0.190 (b)	0.066 (a)

Attributes	p-values	Carrots	Green beans
Sour	0.705	0.033 (a)	0.025 (a)
Delicate	**<0.001**	0.554 (b)	0.041 (a)
Sweet	**0.013**	0.347 (b)	0.207 (a)
Bitter	0.206	0.033 (a)	0.066 (a)

Attributes	p-values	Green beans	Peas
Sour	0.705	0.025 (a)	0.033 (a)
Delicate	1.000	0.042 (a)	0.042 (a)
Sweet	**0.009**	0.208 (a)	0.317 (b)
Bitter	0.564	0.067 (a)	0.050 (a)

Note: different letters indicate a significant difference by Sheskin method, values in bold show a significant difference at $p<0.05$
