# Supplementary material for: Does Responsiveness to Basic Tastes Influence Preadolescents’ Food Liking? Investigating Taste Responsiveness Segment on Bitter-Sour-Sweet and Salty-Umami Model Food Samples
Source: Nutrients. 2021 Aug 7;13(8):2721. doi: 10.3390/nu13082721 (PMC8401806; doi:10.3390/nu13082721)
Supplement: Supplementary file 1 [file nutrients-13-02721-s001.zip › S4 Supplementary Table 4.pdf]

Supplementary Table 4: Norwegian version of the Italian Child Food Neophobia Scale (ICFNS) [64]. (R) indicates the neophilic items for which the score was reversed.

**1. Jeg spiser ofte ny og uvant mat (R)**				
☐ veldig usant	☐ usant	☐ hverken sant eller usant	☐ sant	☐ veldig sant
**2. Jeg stoler ikke på ny mat**				
☐ veldig usant	☐ usant	☐ hverken sant eller usant	☐ sant	☐ veldig sant
**3. Hvis maten er ny for meg, smaker jeg ikke på den.**				
☐ veldig usant	☐ usant	☐ hverken sant eller usant	☐ sant	☐ veldig sant
**4. Jeg liker å prøve rare smaker og matvarer som er uvanlige og kommer fra ulike land (R)**				
☐ veldig usant	☐ usant	☐ hverken sant eller usant	☐ sant	☐ veldig sant
**5. Når jeg spiser middag hos en venn, liker jeg å smake ny mat (R)**				
☐ veldig usant	☐ usant	☐ hverken sant eller usant	☐ sant	☐ veldig sant
**6. Jeg er redd for å smake på mat jeg ikke har prøvd før**				
☐ veldig usant	☐ usant	☐ hverken sant eller usant	☐ sant	☐ veldig sant
**7. Jeg er veldig kresen angående mat.**				
☐ veldig usant	☐ usant	☐ hverken sant eller usant	☐ sant	☐ veldig sant
**8. Jeg spiser hva som helst! (R)**				
☐ veldig usant	☐ usant	☐ hverken sant eller usant	☐ sant	☐ veldig sant
